# Residents’ perspective on duty hours at an Israeli tertiary hospital

**DOI:** 10.1186/s13584-022-00521-0

**Published:** 2022-02-10

**Authors:** Elad Apt, Tslil Regev, Jacob Shapira, Ori Haberfeld, Ori Samuel Duek, Ronen Bar-Yoseph

**Affiliations:** 1 Orthopedic Surgery Division, Rambam Health Care Campus, Haifa, Israel; 2 Ruth Rappaport Children’s Hospital, Rambam Health Care Campus, Haifa, Israel; 3 Department of Thoracic Surgery, Rambam Health Care Campus, Haifa, Israel; 4 Plastic and Reconstructive Surgery Department, Rambam Health Care Campus, Haifa, Israel; 5 Pediatric Pulmonology Institute, Ruth Rappaport Children’s Hospital, Rambam Health Care Campus, Haifa, Israel

**Keywords:** Shifts, Residents, Night floats, Questionnaires

## Abstract

**Background:**

Specialization in medical professions is considered a challenging and intensive period due to the number and sequence of duty hours. Considering the effect of duty hours on residents, both physically and mentally, several models have been created over the years to address this complexity. The two main model schools aim to decrease the duty hour length and night shift (i.e., night float, NF) frequency. In recent years, duty hours have become a source of disagreement and frustration among the medical community, both residents and attendings. A possible change in the duty hour structure may affect residents in terms of several parameters, such as patient safety, the well-being of the physician and the degree of training of the resident.

**Purpose:**

(1) To investigate medical residents’ perspectives on their duty hours utilizing online questionnaires on their effect on the work environment and (2) to assess residents’ preferences in relation to the suggested shortened shift and NF models.

**Methods:**

Questionnaires were emailed to all residents (main residents and fellows) at an Israeli tertiary medical center between March 2020 and April 2020. Questions were scored from 1 (disagree) to 5 (fully agree).

**Results:**

Two hundred and sixty residents (227 main residents, 43 fellows) participated in the study (40% female). The score for the degree of balance between work and personal life was low (0.9±1.99). The shortened shift model was perceived by the residents as more compatible with a balanced lifestyle than the NF model (3.77 ± 1.20 and 3.14 ± 1.26, respectively, P < 0.0001). Neither model was considered to risk impairing professional training (2.33 ± 1.45 and 2.47 ± 1.25, respectively, P = 0.12). Overall, 74% of the residents were not willing to lower their income if the decision were made to change models, and 56% were not willing to increase the number of shifts.

**Conclusions:**

There is agreement among residents that shortening shift hours to 16 h would have a positive effect on the balance between personal life and work. In the eyes of residents, the change would not impair their training during residency.

**Supplementary Information:**

The online version contains supplementary material available at 10.1186/s13584-022-00521-0.

## Background

The residency period is a cornerstone in physician training after medical school in the process of becoming experts in various medical professions. During this period, physicians acquire the clinical experience required for their work as specialist physicians under the supervision of senior physicians.

Residency is composed of daily work as well as shifts that usually last from the end of the workday until the next morning, adding to a total of 26 consecutive hours in the hospital. This model has several advantages and disadvantages. At times when the presence of senior physicians is relatively low, shifts provide a more "independent" environment than regular duty hours, when senior physicians are on call on demand. On the other hand, sleep deprivation as a result of long duty hours may impair residents' ability to withstand the acceptable standard of care as well as to efficiently absorb and improve their clinical skills. In addition, due to absences following duty hours, residents may decrease their exposure to supervised clinical work, both at the outpatient clinic and in the operating theater [1].

The demanding workload along with the desire for clinical exposure may result in suboptimal management of cases during shift hours. The event that led to the collapse of the traditional residency model occurred in 1984 in the state of New York (USA), when a student named Libby Zion was hospitalized at Weil Cornell Medical Center after taking antidepressant products. Due to poor diagnosis and management, the patients’ condition deteriorated, and the patient eventually died as a result of a heart attack [2]. The court attributed the tragic outcome to the fact that two residents were in charge of 40 patients for a long period of time; after this case, one of the recommendations was that residents could not work more than 80 h a week or more than 24 consecutive hours [3]. There are various models around the globe for specialization in medical professions. In Israel, a collective agreement was signed in 2000 that reduced shift hours from 32 consecutive hours with regular work the next morning to 26 h in addition to a day off after a shift [4]. In the collective agreement signed in 2011, a weekly rest day was provided in case the schedule did not allow it during a given week [5]. In addition, each resident has 22 vacation days per year, and 30 days before each board exam as protected time during the residency. Furthermore, following a legal hearing in 2014, the Tel Aviv Regional Labor Court recommended that sleep breaks should be set between 2 and 4 h on physicians' shifts [6] Israel’s law regarding work and rest hours limits the number of duty hours per week and sets a mandatory minimum of weekly rest hours, but occupations that involve treating people could be excluded from these terms according to the language of the law [7].

Similarly, in the United States and Canada, there are restrictions on both shift hours and weekly work hours among residents. In Europe, there is a limit under the European Working Time Directive (EWTD) to 48 working hours per week [8]. In contrast, the model used in Australia does not limit working hours among residents. Notably, the differences among countries are also manifested in their expected compliance with such laws [9].

To date, despite the active discussion among the community of physicians and the general public, there is no consensus on how duty hours affect patients’ safety, physicians' well-being and training of residents [1, 10, 11]. A recent article in the New England Journal of Medicine (NEJM) reported a clinical trial comparing 2 schedules for pediatric residents during their intensive care unit (ICU) rotations: extended duration work schedules that included shifts of ≥ 24 h or schedules of 16 h or less. The study concluded that in contrary to the authors hypothesis, an increase in the number of critical errors was found after a switch to 16-h shifts. [12].

To the best of our knowledge, there are no studies reporting residents' opinions regarding the possible impact of shifts during residency on personal and professional life and their opinions about possible changes. The purpose of the study is to investigate residents’ opinions on various shift-related issues and to change the existing shift model.

## Methods

In May 2020, a questionnaire was sent to all specializing physicians in Rambam Medical Center. Inclusion criteria included physicians who were in the course of their residency or subspecialty fellowship and performed shifts as part of their residency duties.

A digital questionnaire was sent (via e-mail with a link to Google Forms) to the study group (320 residents and subspeciality fellowship); it collected data on gender, marital status, completion of residency board exams, average number of shifts per month, average number of hours of sleep during shifts, frequency of being asked to stay to complete tasks after shifts and number of shifts during the weekend on average. In addition, the resident was asked to rate the tested models on a number of parameters in addition to providing optional suggestions. The models tested were: shortening duty hours to 16 h (e.g., 4:00PM–8:00AM every few night) or working night floats (NF); working several consecutive nights a month, with the work being in the mornings the rest of the time (e.g., in one department NF could be 3 consecutive nights, each shift 16 h long, once every 4 weeks and in a different department it could be 7 consecutive nights for 12 h, once every 6 weeks). To note, the night shift model options were not connected to a direct financial outcome. Rather, the residents were asked regarding the financial topic in a separate direct question.

During the week following the distribution of the questionnaire, an additional reminder was sent to the resident's e-mail, and the authors contacted the residents directly in the various departments.

For each question, the residents had to indicate their opinion by marking the most suitable answer on a 1–5 scale (1 = fully agree, 5 = fully disagree).

### Statistical methods

Descriptive statistics including the means, standard deviations, medians, ranges, and percentages were calculated for all variables in the study. The examination of the balance between personal life and work in the two proposed models (duty hours shortened to 16 h or NFs) was calculated using a paired t-test. Significance was considered when p < 0.05. Data processing was performed in SPSS software version 25.

## Results

### Demographics and current status

**Fig. 1:**
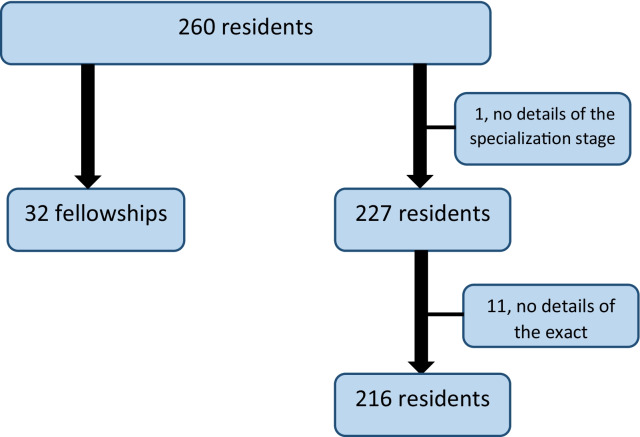
Summary of all respondents to the questionnaire (Additional File 1)

Two hundred and sixty residents (81% of the total residents performing night shifts) answered the survey (Fig. 1). Overall, two hundred twenty-seven of them (87%) were in a primary residency. Of them, 63% had not yet taken the first part of their board exam, 59% were males and 62% were both in a relationship and had children. Additionally, 12% of the respondents were completing subspecialty fellowships. One participant was excluded due to missing data regarding the specialization stage. Eleven of the residents in the primary residency were not included in some of the statistical analysis because they only partially filled out the survey (Table 1, Fig. 1).

**Table 1:** Demographic details and overview of the current situation

	%	N
Phase in residency*		
Pre board exam A	63%	163
Post board exam A	25%	64
Fellowship	12%	32
Gender		
Females	40%	104
Males	58%	152
Marital status		
In a relationship with children	61%	159
In a relationship without children	20%	52
Single	17%	45
Single parent	< 1%	2
Average number of shifts per month		
< 4	8%	22
4	13%	35
5	32%	82
6	37%	95
7	9%	23
8 +	< 1%	2
How long normally sleep-break lasts during night shifts?		
No sleep-breaks	13%	35
Under 1–2 h	38%	97
Between 1–2 h	34%	89
Over 1–2 h	15%	38
How often do you remain at the hospital for different assignments following the night shift?		
Never	27%	69
Rarely	21%	55
Sometimes	26%	67
Usually	26%	68
Average number of weekend shifts per month		
0	4%	10
1	17%	43
2	71%	185
3 +	8%	21

*During residency in Israel, residents take two exams (A and B) to be certified as Specialists in their field

At the time of the study, 89% of the hospital residents had worked up to 6 shifts a month. Only 15% of the residents indicated that they slept more than two hours on average per shift. Twenty-six percent indicated that they frequently stayed beyond the 26 h to perform additional departmental tasks, while another 26% indicated that they were not required to do so at all (Table 1).

### Residents’ opinions

The balance between work and personal life received a low score (1.99 ± 0.9). A preference was found among residents for the model of shortening the shift length to 16 h. The residents thought that compared to NFs, this model would improve the balance between personal life and work (3.77 ± 1.20 and 3.14 ± 1.26, respectively, P < 0.0001). Overall, the residents did not perceive that either model would significantly impair training, with no significant difference between the models (2.33 ± 1.45 and 2.47 ± 1.25, respectively). Effect of seniority (phase of residency; before and after phase A) was found between the groups and results were affected in both models (Table 2).

**Table 2:** Summary of the effect of the different models on the parameters tested

	16-h shifts	Night floats (NFs)	P-value
Balance between personal life and work	1.20 ± 3.77	1.26 ± 3.14	p < 0.0001
	M 3.8 ± 1.2	M 3.16 ± 3.16	
	F 3.71 ± 1.22	F 3.06 ± 1.22	
	P = 0.59	P = 0.53	
	Before 1.12 ± 4.00	Before 1.27 ± 3.19	
	After 1.30 ± 3.48	After 1.24 ± 3.05	
	P = 0.003	P = 0.42	
Impaired resident training	1.25 ± 2.47	1.45 ± 2.33	p = 0.12
	M 2.45 ± 1.50	M 2.51 ± 1.31	
	F 2.12 ± 1.36	F 2.43 ± 1.18	
	P = 0.079	P = 0.59	
	Before 1.39 ± 2.16	Before 1.19 ± 2.30	
	After 1.53 ± 2.60	After 1.32 ± 2.78	
	P = 0.017	P = 0.003	

M, males; F, females; Before, before phase A; After, after phase A + fellows

Among the major surgical departments (general surgery, orthopedics, gynecology, neurosurgery, and anesthesia), the residents did not report that the 16-h shortened shift model would significantly impair training in comparison to the NF model (1.86 ± 1.52 and 2.32 ± 2.02, respectively, P = 0.014).

**Fig. 2:**
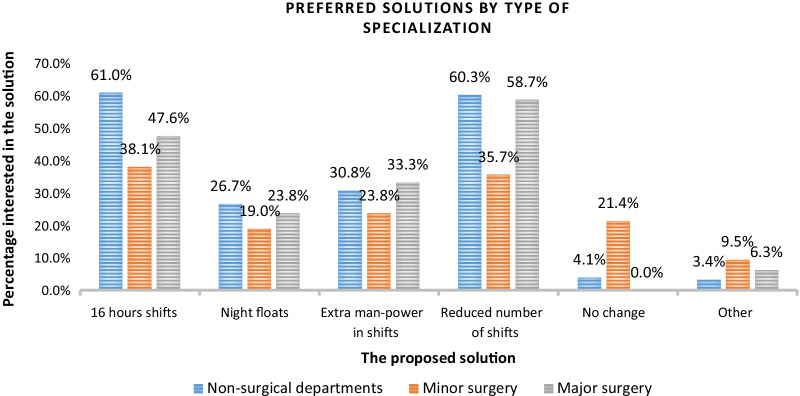
Division by specialization group regarding the proposed models (Additional File 2). (Major specializations: General surgery, Gynecology, Orthopedics, Neurosurgery and Anesthesia. Minor specializations: Nose and throat, Ophthalmology, Plastic surgery, Chest surgery, Cardiac surgery)

Ninety-two residents (35%) did not support either the model for 16-h shortened shifts or the NF model. Among this group, 64% were interested in additional manpower during shifts, 33% of them wanted to reduce the number of shifts, and only 17% answered that there was no need to change the current situation. Of these residents, only 28% had not yet entered the first stage of the residency board exam. In addition, 37% of these residents were completing a surgical residency (Fig. 2).

Regarding the effects of changing the current situation, 77% of the residents indicated that there was room for additional residents in their department to change the existing model. Seventy-four percent were not prepared to lower their income if the decision were made to change models (no gender effect), and 56% were not prepared to increase the number of shifts. In addition, the residents were not willing to extend the residency in exchange for changing the model (score of 1.75 ± 1.08).

The residents perceived the workload to be more of a factor than the number of shifts in making the residency difficult (3.1 ± 1.3 compared to 1.9 ± 1.2). Regarding whether a decrease in the number of shifts would improve working conditions, the residents thought it would only moderately improve conditions (3.07 ± 1.32).

## Discussion

The aim of the study was to examine residents' opinions by surveying residents and fellows from various medical fields and examining their perspective of two models (i.e., shortened 16-h shifts and NFs). The survey was conducted in a tertiary hospital, and most hospital residents agreed to share their opinions (81%). It was found that the residents perceived that implementing the shortened shift model (16 h) or the NF model would lead to an improvement in personal life-work balance and would not impair the degree of training as a resident. Shortening duty hours was recognized as a model that would support a balanced lifestyle, more so than the NF model. While 64% of the residents were interested in shortening shifts, most residents (77%) were not interested in a subsequent reduction in their salaries as a result of a change in the current shift format. Fifty-six percent were not willing to increase the number of shifts in exchange for a change in the existing model.

A study performed on retrospective data after a change of models was made (post hoc) in specific specialties showed concern among general practitioners (GPs) when work hours were limited according to guidelines published in 2011 in Accreditation Council for Graduate Medical Education (ACGME) [13]. Another work published by Kelly et al. [14] described concern among surgeons regarding harm to their training after limiting shift hours and weekly hours.

### Current situation

In 2011, a new collective agreement was signed between the government and physicians in which residents' positions were added to all hospitals in the country to reduce the number of residents’ shifts to a maximum of 6 per month [15]. Indeed, in our study, 89% of residents reported that they worked up to 6 shifts per month, as targeted in the agreement. Although the Tel Aviv Regional Labor Court ruled in 2014 that sleep breaks should be at least two hours per night shift (preferably 2–4 h) [6], only 15% of residents stated that they slept two hours a night on average, while the rest stated that they often did not sleep as many hours as required. Notably, based on the survey, even adhering to the required sleeping hours will not obviate the need to change the shift method.

The residents perceived workload to be an even more important factor than the number of shifts in making the residency difficult (3.1 ± 1.3 compared to 1.9 ± 1.2). One of the optional measures to both improve the workload and potentially increase the rest time per shift is to reduce "paramedical" work, which in the residents' opinion moderately affects the workload on duty (3.4 ± 1.2).

### Shortening shifts

Many studies have tested various parameters when changing the working method of residents [1]. The main parameters that have been examined are the impact on patient safety, well-being of the therapist and degree of training of the physician.

A review article published in 2014 examined the relationship between the duration of shifts and patient-related parameters such as mortality or complications, health-related parameters such as length of hospitalization and caregiver health-related parameters. The duration of the shifts tested ranged from 13 to 36 h. Out of the 13 jobs examined, one job showed an increase in the rate of complications in shifts lasting over 16 h. Two additional studies found a shorter hospitalization duration in favor of the shorter shift group, and two additional studies showed improvement in the quality of life of the physicians in this group. Seven additional studies did not find a difference between the groups in relation to the different indices [1]. Another recent study published in the NEJM examined patient safety in the intensive care department and found that there was an increase in the number of critical errors (defined as errors that resulted in patient injury or high-risk patient injury) after switching to shorter shifts [12]. The findings from this work reinforce the articles cited above. From the survey conducted in the current study, it seems that there is a preference for shorter shifts than the currently used shifts (Table 2).

Another parameter that has been examined in previous works is the degree of impact on residents’ training and participation in academic activities. In the review article cited above, in 5 out of 8 cases, there was an impairment in the training of the resident, which was reflected in a decrease in the number of working hours with senior physicians, participation in academic activities and number of surgery sessions. The conclusion of the previous article is inconsistent with our study, in which the residents perceived that the degree of impairment in training after switching to shortened shifts would be low. However, seniority in residency affected the perception toward an impairment in residency training and balance between personal life and work (Table 2) [1].

### Night floats

Bolster et al. [1] published a review that included studies that examined shift work under the NF framework. Six out of ten studies found a negative impact on the therapist's well-being. The deterioration in well-being was manifested in decreased sleep hours, increased fatigue, and increased stress. Two studies examined the effect on patient safety: one showed a decrease in diagnostic errors, while the other showed no difference in metrics such as length of stay, rehospitalizations and mortality [1].

Two additional studies examined the effect on the caregiver's health when setting rest hours on duty: one showed improvement in quality of sleep and duration of sleep, while the other showed no difference. These findings are inconsistent with the residents' perception in our study that working under such a model would improve quality of life.

Four out of 6 studies on the NF model showed a decrease in the quality of training. Our work showed a perception that changing to other models would not necessarily affect the training, which does not correspond with the findings of most of the published works.

Notably, several studies have been published that suggested that the physician’s well-being is lower under the NF model than under standard shifts with one shift every few days [16, 17]. Kelly et al. [14] also showed that under the NF model, specialized surgeons lose many hours of operating room time than when they have one shift every few days.

Over the last few years, the Israeli Medical Association (IMA) has been subjected to structural changes. Whereas in the past, decision making has been solely conducted by senior physicians and governmental personnel, recently residents have been introduced into IMA to proactively advance burning topics. These steps led to a position paper describing possible alternatives to the night shift model. As part of the residents’ struggle, in October 2021 many have signed their resignation as an act of disapproval against the burden of the long hour shifts, hoping that this act will encourage the ministry of health to move faster toward a new residency course. All the above events have renewed the discussion on this topic with some opponents questioning the benefit of changing the current format and expressing their concerns for the next generation of attending physicians in the post-night shifts era. This study presents real life insight to the resident’s perspective in a tertiary hospital in Israel and might add relevant data to this debate.

This work has a number of limitations. First, the responses were collected at a certain time point and not over time; therefore, specific events (such as the beginning of the COVID-19 pandemic) might have had a large effect on the residents’ responses. Another limitation is that the survey was conducted in only one hospital in northern Israel, while there may be differences among different parts of the country or among different health systems in different places in the world. Not all the optional night shift models were assessed in the questionnaire. Israel Medical Association (IMA) published on June 2021 a statement regarding optional night shift models. To note, in this document the first two options were presented: up to 15 (9:00PM-12:00PM) or up to 18 h (04:00PM-08:00AM) and the third was night floats version (18 h per shift and followed by 30 h rest) for a set period of time. Due to these time discrepancies, the options in our study were similar but not identical to IMA’s.

In conclusion, shortening shifts to 16 h is the preferred model for residents in medical professions and is perceived as having the potential to improve the balance between work and personal life without significantly impairing the degree of training. Most residents are not willing to reduce their income or increase the number of shifts in exchange for changing the existing shift model. Larger studies that include multiple hospitals as well as evaluations of residents’ opinions before and after different changes are warranted.

## Supplementary Information

**Additional file 1: Figure 1**. Summary of all respondents to the questionnaire.

**Additional file 2: Figure 2**. Divison by specialization group regarding the proposed models.

## Data Availability

The datasets used and/or analyzed during the current study are available from the corresponding author upon reasonable request.

## Abbreviations

NF: Night float
EWTD: European Working Time Directive
NEJM: New England Journal of Medicine
ACGME: Accreditation Council for Graduate Medical Education
M: Males
F: Females
