# Supplementary material for: Residents’ perspective on duty hours at an Israeli tertiary hospital
Source: Isr J Health Policy Res. 2022 Feb 10;11:11. doi: 10.1186/s13584-022-00521-0 (PMC8830127; doi:10.1186/s13584-022-00521-0)
Supplement: Supplementary file 1 — Additional file 1: Figure 1. Summary of all respondents to the questionnaire. [file 13584_2022_521_MOESM1_ESM.docx]

**Figure 1.** Summary of all respondents to the questionnaire

260 residents

1 , no details of the specialization stage

227 residents

32 fellowships

11, no details of the exact residency

216 residents
