# Supplementary material for: Residents’ perspective on duty hours at an Israeli tertiary hospital
Source: Isr J Health Policy Res. 2022 Feb 10;11:11. doi: 10.1186/s13584-022-00521-0 (PMC8830127; doi:10.1186/s13584-022-00521-0)
Supplement: Supplementary file 2 — Additional file 2: Figure 2. Divison by specialization group regarding the proposed models. [file 13584_2022_521_MOESM2_ESM.docx]

**Figure 2.** Division by specialization group regarding the proposed models.
